# Supplementary material for: Mercury Content and Amelioration of Its Toxicity by Nitric Oxide in Lichens
Source: Plants (Basel). 2023 Feb 7;12(4):727. doi: 10.3390/plants12040727 (PMC9967695; doi:10.3390/plants12040727)

Supplementary materials for the work:

Mercury Content and Amelioration of Its Toxicity by Nitric Oxide in Lichens

Jozef Kováčik [1,*], Lenka Husáková [2], Martina Piroutková [2], Petr Babula [3]

[1] Department of Biology, University of Trnava, Priemyselná 4, 918 43 Trnava, Slovak Republic
[2] Department of Analytical Chemistry, Faculty of Chemical Technology, University of Pardubice, Studentská 573 HB/D, 532 10 Pardubice, Czech Republic
[3] Department of Physiology, Faculty of Medicine, Masaryk University, Kamenice 753/5, 625 00 Brno, Czech Republic
* Correspondence: jozkovacik@yahoo.com (J.K.); Tel.: +421-33-5939-519

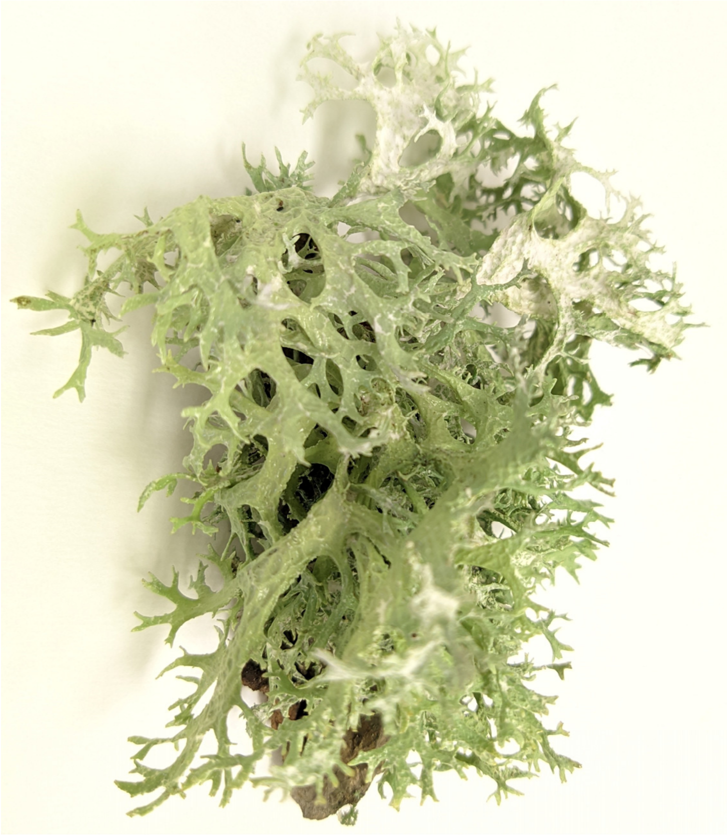

Supplementary Figure S1. Representative photo of the lichen *Evernia prunastri* used for the physiological experiment with exogenous Hg and/or NO application.

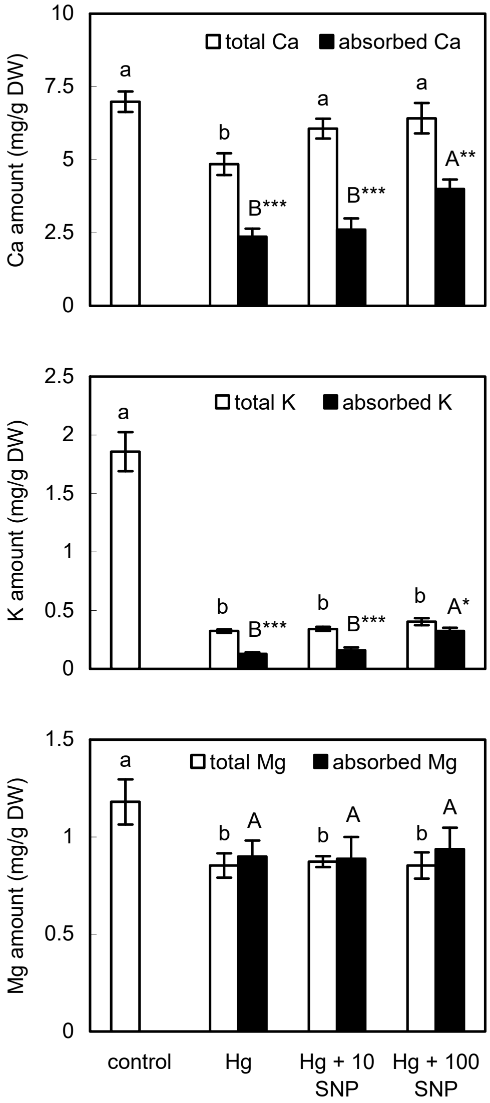

Supplementary Figure S2. Amount of selected macronutrients in the lichen *Evernia prunastri* after 24 h of exposure to 100 μM Hg with the addition of 10 or 100 μM NO donor (SNP). Control means no addition of Hg or SNP. Data are means ± SDs shown as bars (n = 3). Values for each fraction (total or absorbed), followed by the same letter(s), are not significantly different according to Tukey's test (P<0.05). *, ** and *** indicate significant difference between total and absorbed fraction in the given treatment at 0.05, 0.01 or 0.001 level of Student's t-test. Absorbed content means that thalli were rinsed with Na_2EDTA to remove surface-bound metals.

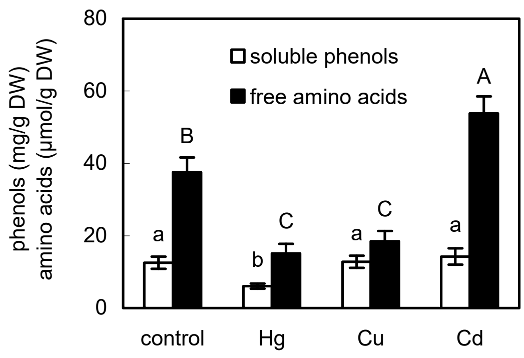

Supplementary Figure S3. Impact of 100 µM Hg, 100 µM Cu or 100 µM Cd (all cations as chlorides) on the amount of soluble phenols and free amino acids in the lichen *Evernia prunastri* after 24 h of exposure. Control means only HEPES buffer without any metal. Data are means ± SDs shown as bars (n = 3). Values, followed by the same small or capital letter, are not significantly different according to Tukey's test (P<0.05).

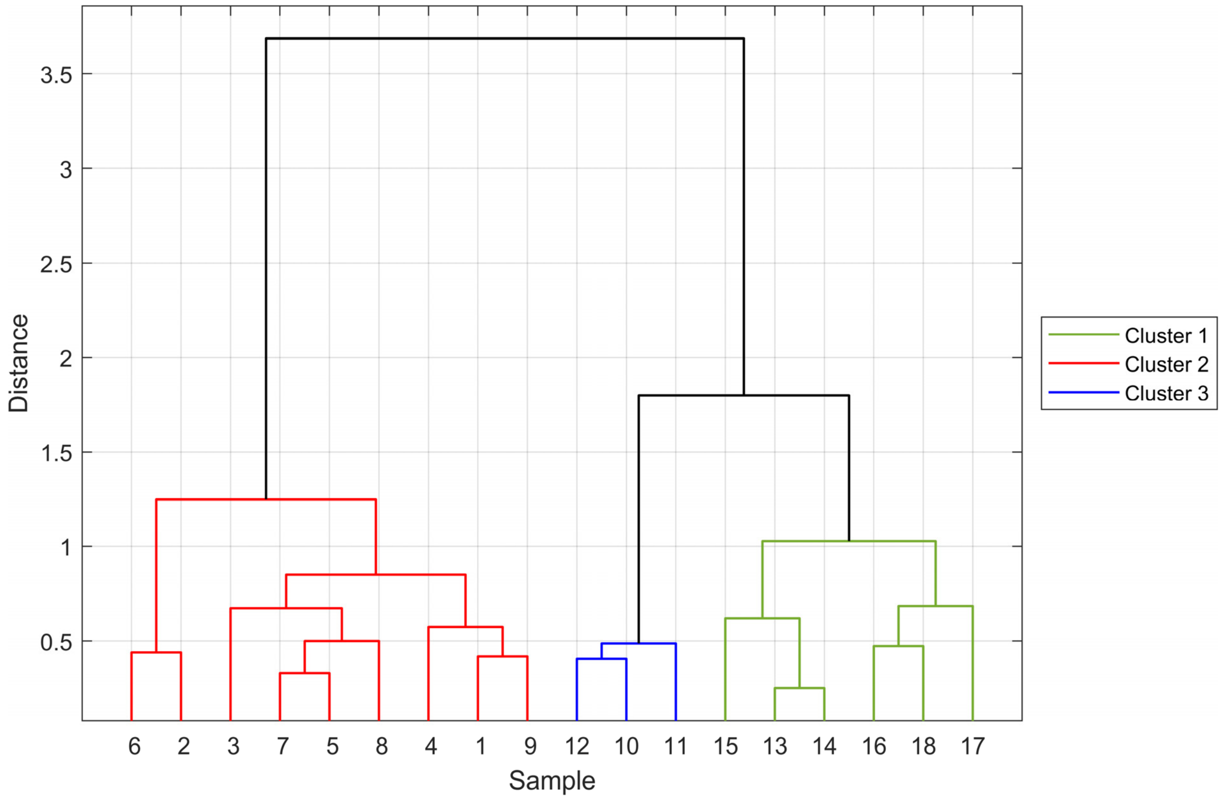

Supplementary Figure S4. Cluster analysis dendrogram of individual samples of the lichen *Evernia prunastri* exposed for 24 h to 100 µM Hg or no Hg with or without co-application of NO donor sodium nitroprusside (SNP). Numbers indicate individual samples (1 – 3: -Hg/0 SNP, 4 – 6: -Hg/10 SNP, 7 – 9: -Hg/100 SNP, 10 – 12: +Hg/0 SNP, 13 – 15: +Hg/10 SNP, 16 – 18: +Hg/100 SNP). Note separation of samples without Hg (no. 1 – 9) from Hg-treated samples (no. 10 – 18) and also separation of +Hg samples without SNP (no. 10 – 12) or with SNP (13 – 18).

Supplementary Table S1. Pearson's correlation analysis between parameters in the lichen *Evernia prunastri* after 24 h of exposure to 100 µM Hg with the addition of 10 or 100 µM NO donor (SNP) plus control: *for the correlation, only total fraction of elements (including control with no Hg/SNP)* was paired with the respective sets of metabolites. Hg content was not included in correlations because its amount in control thalli was much lower compared to exogenous Hg application (see next Supplementary Table S2 for correlation of absorbed Hg with other parameters and main text for quantity of Hg in lichen thalli). * The corresponding correlation in R is considered significant as an off-diagonal element of P was smaller than the significance level of 0.05. Green numbers indicate significantly positive values (no negative correlation was observed). AsA – ascorbic acid, NPT – non-protein thiols, SP – soluble phenols, FAA – free amino acids.

	Ca	K	Mg	AsA	NPT	SP	FAA
Ca	1						
K	0.6379*	1					
Mg	0.6297*	0.8742*	1				
AsA	0.6500*	0.9244*	0.8613*	1			
NPT	0.7731*	0.7284*	0.6182*	0.7694*	1		
SP	0.5886*	0.9671*	0.9364*	0.9149*	0.6302*	1	
FAA	0.1936	0.8357*	0.8037*	0.7570*	0.3437	0.8817*	1

Supplementary Table S2. Pearson's correlation analysis between parameters in the lichen *Evernia prunastri* after 24 h of exposure to 100 µM Hg with the addition of 10 or 100 µM NO donor (SNP): *for the correlation, only absorbed fraction of elements (including Hg, Hg+10 SNP and Hg+100 SNP)* was paired with the respective sets of metabolites. * The corresponding correlation in R is considered significant as an off-diagonal element of P was smaller than the significance level of 0.05. Green and red numbers indicate significantly positive or negative values, respectively. AsA – ascorbic acid, NPT – non-protein thiols, SP – soluble phenols, FAA – free amino acids.

	Hg			
Hg	1	Ca		
Ca	-09036*	1	K	
K	-0.8850*	0.9618*	1	Mg
Mg	-0.4087	0.4465	0.3736	1
AsA	-0.8688*	0.8555*	0.8475*	0.1517
NPT	-0.4762	0.6607	0.6577	0.0162
SP	0.3787	-0.5028	-0.3048	-0.6265
FAA	0.4377	-0.6037	-0.6658	-0.0943

Supplementary Table S3. Heat map of the analyte loadings on the two components for PCA graph in the Fig. 4 of the main text. SP (soluble phenols), FAA (free amino acids), NPT (non-protein thiols), AsA (ascorbic acid).

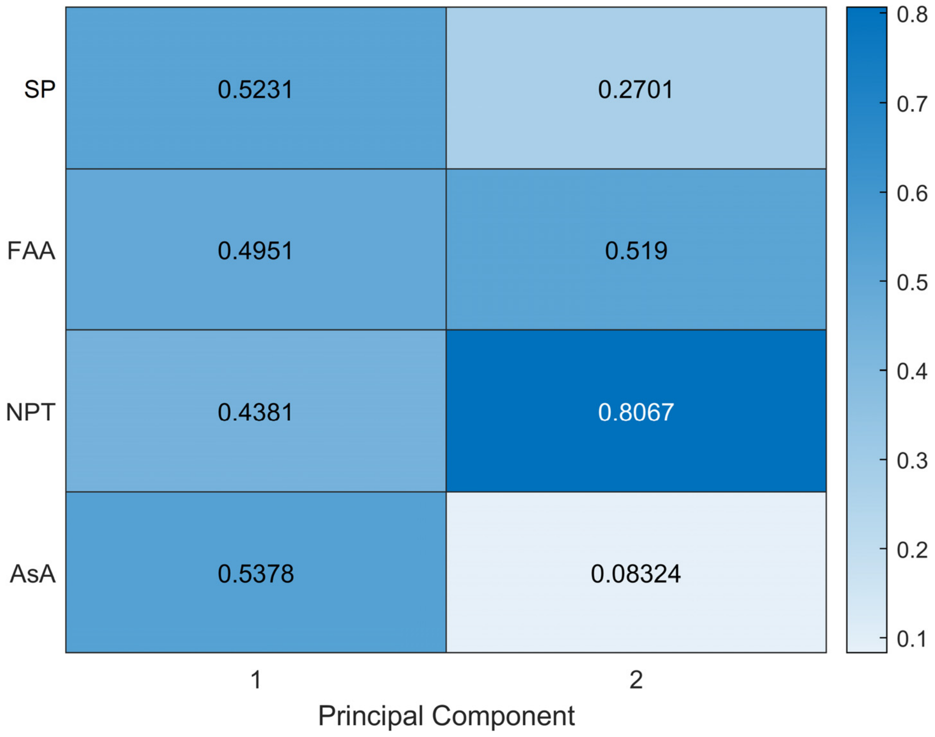

Supplement: Supplementary file 1 [file plants-12-00727-s001.zip › plants-2161727-supplementary.pdf]
